# LGI1 Antibody-Associated Encephalitis Complicated with Sjögren's Syndrome and Acute Cerebral Infarction: A Case Report and Literature Review

**DOI:** 10.2174/0118715303410062250729095927

**Published:** 2025-11-20

**Authors:** Dongxiao Jiang, Yanfen Lian, Xia Zhan, Jingjing Wang, Pengjiao Xu, Yuhua Bi

**Affiliations:** 1 Department of Neurology, Weihai Central Hospital Afffliated to Qingdao University, Weihai, 264400, China

**Keywords:** Autoimmune encephalitis, LGI1 antibody, acute cerebral infarction, Sjögren's syndrome, faciobrachial dystonic seizures (FBDS), nervous system

## Abstract

**Background:**

Anti-LGI1 antibody-associated encephalitis is a rare autoimmune neurological disorder, and primary Sjögren's syndrome is a systemic autoimmune disease with multisystem involvement. The coexistence of these conditions with acute cerebral infarction is extremely rare and highlights complex interactions between autoimmune and vascular mechanisms.

**Case Presentation:**

This case report describes a middle-aged female patient diagnosed with LGI1 antibody-associated encephalitis, complicated by Sjögren's syndrome and acute cerebral infarction. The patient presented with an acute onset of symptoms, including delayed response, cognitive impairment, and right-sided weakness. Brain MRI revealed a fresh infarction in the left basal ganglia region. Blood and cerebrospinal fluid tests confirmed the presence of LGI1 antibodies, with blood anti-SS-A antibody levels at +++ and anti-SS-B antibody levels at +, as well as anti-Ro-52 antibody levels at +++.

**Conclusions:**

This report emphasizes the importance of recognizing the overlap between these conditions, as timely diagnosis and intervention can lead to better outcomes in similar cases. By examining the relationship between anti-LGI1 antibody-associated encephalitis, primary Sjögren's syndrome, and acute cerebral infarction, this paper aims to expand the understanding of the complex interactions between autoimmune encephalitis and systemic disorders.

## 1 INTRODUCTION

Anti-leucine-rich glioma-inactivated 1 (LGI1) encephalitis is a common type of autoimmune encephalitis. Its typical clinical manifestations include memory impairment, faciobrachial dystonic seizures (FBDS), confusion or psychiatric disorders, and hyponatremia [1]. Primary Sjögren's syndrome, a chronic inflammatory autoimmune disease, can affect multiple parts of the nervous system. When the central nervous system is involved, it may present with cognitive dysfunction, ischemic stroke, demyelinating diseases, and other symptoms [2]. Both LGI1 antibody-associated encephalitis and Sjögren's syndrome are autoimmune diseases and can occur concurrently, although the combination with cerebral infarction is rare in clinical practice [3]. Herein, we reported a case of a patient who presented with memory impairment and psychiatric abnormalities. During the course of the disease, the patient developed limb weakness. After thorough examination, the patient was diagnosed with LGI1 antibody-associated encephalitis complicated by Sjögren's syndrome and cerebral infarction. Following treatment, the patient's symptoms alleviated.

## 2 CASE PRESENTATION

**Fig. (1):**
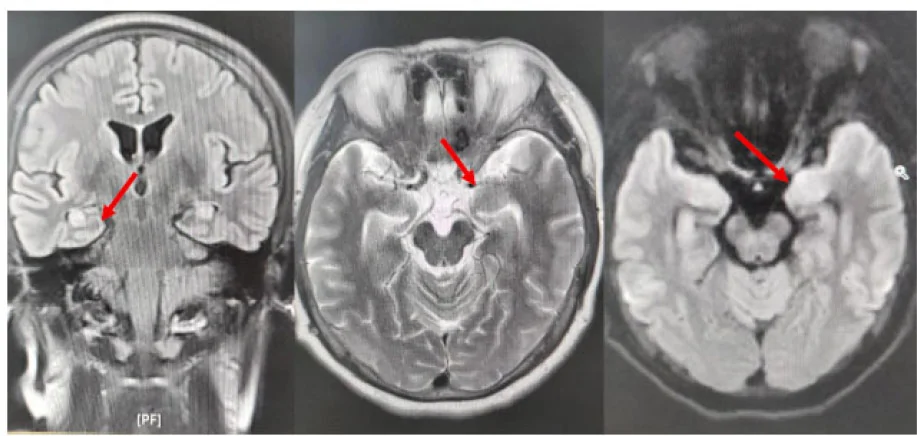
Brain MRI shows bilateral temporal lobe hippocampi with high T2WI signal intensity and blurred margins. FLAIR imaging shows increased signal intensity, more pronounced on the left side.

A 50-year-old woman presented with a one-month history of worsening memory impairment, which was exacerbated over the preceding two weeks by progressive cognitive abnormalities. She was admitted to the local hospital on June 11, 2021 with no significant past medical history. Throughout the disease course, the patient did not exhibit unilateral limb convulsions or involuntary tremors. Autonomic symptoms, such as hyperhidrosis, piloerection, or disturbances in urination and defecation, were absent. Neurological examination revealed clear consciousness but marked cognitive abnormalities, including an inability to respond correctly to questions and non-cooperation with advanced cognitive tests. There were no cranial nerve deficits, and voluntary movements were intact in all four limbs with normal muscle tone. The patient was uncooperative during finger-to-nose and heel-to-shin tests, but there were no pathological signs, and meningeal signs were absent. At admission, laboratory investigations revealed serum sodium levels of 123 mmol/L and chloride levels of 92.2 mmol/L. Tumor markers were within normal limits. Cerebrospinal fluid (CSF) analysis showed a white blood cell count of 2×10^6^/L, total protein of 373.7 mg/L, chloride of 108.8 mmol/L, and glucose of 4.63 mmol/L. Tests for tuberculosis antibodies and cryptococcus antigens were negative in the CSF. Paraneoplastic syndromes panels, including tests for eight specific antibodies, were negative (conducted at Jinan Jinyu Medical Laboratory). Testing for MOG, AQP4, and GFAP antibodies in both serum and CSF also showed negative results (conducted at Jinan Jinyu Medical Laboratory). However, the serum autoimmune encephalitis panel showed elevated LGI1 antibody titers of 1:100, which were confirmed in the CSF (conducted at Jinan Jinyu Medical Laboratory). Electrophoresis of both serum and CSF showed no oligoclonal bands. Brain MRI revealed bilateral hippocampal swelling in the temporal lobe, with patchy areas of slightly low T1-weighted and high T2-weighted signals, as well as blurred margins and increased FLAIR signals, more pronounced on the left side (Fig. **1**). No abnormalities were observed on video electroencephalogram (video EEG) examination.

Chest CT scan showed no abnormalities. Ultrasound examinations of the liver, gallbladder, pancreas, spleen, and urinary system showed no abnormalities, and gynecological ultrasound was unremarkable. Based on these findings, a diagnosis of anti-LGI1 encephalitis was considered. The patient was treated with intravenous methylprednisolone (1000 mg for 3 days, followed by 500 mg for 3 days, and 250 mg for 3 days). After treatment, the patient's psychiatric symptoms disappeared, and a significant memory improvement was noted, with the Montreal Cognitive Assessment (MoCA) score rising to 25. She was subsequently transitioned to oral prednisolone (60mg) and discharged, with a steroid tapering regimen of 5 mg every two weeks until discontinuation, resulting in complete resolution of symptoms.

On March 22, 2022, the patient was readmitted due to two days of delayed response and altered mental status, following an argument with her family. Two days prior, she had developed delayed response and tangential speech. Twelve hours before admission, she developed right-sided weakness, difficulty holding objects in her right hand, and a tendency to veer to the right while walking. She did not experience slurred speech, choking while drinking, or difficulty swallowing. There were no limb convulsions, no involuntary limb tremors, no excessive sweating, no piloerection, and no abnormalities in urination or defecation. Despite no improvement at home, she sought medical attention. Upon admission, her blood pressure was 128/90 mmHg. She was conscious but exhibited delayed responses and reduced cognitive function, with a Montreal Cognitive Assessment (MoCA) score of 6. Extraocular movements were intact without nystagmus. Pupils were equal and reactive to light, measuring approximately 3mm in diameter. Facial features were symmetrical with a midline tongue and uvula elevation. Right-sided muscle strength was graded as 4/5, with right-hand grip strength of 2/5, and no sensory deficits. Coordination was normal, but brisk tendon reflexes (++), along with positive pathological reflexes on the right side, were noted. The neck was supple with no resistance.

**Fig. (2):**
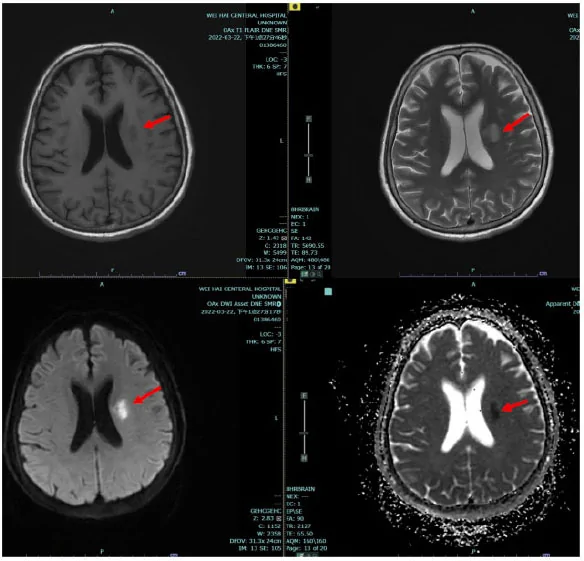
Brain MRI from March 22, 2022, shows patchy areas of prolonged T1 and T2 signal intensities in the left basal ganglia, with increased signal on DWI and low signal on ADC.

**Fig. (3):**
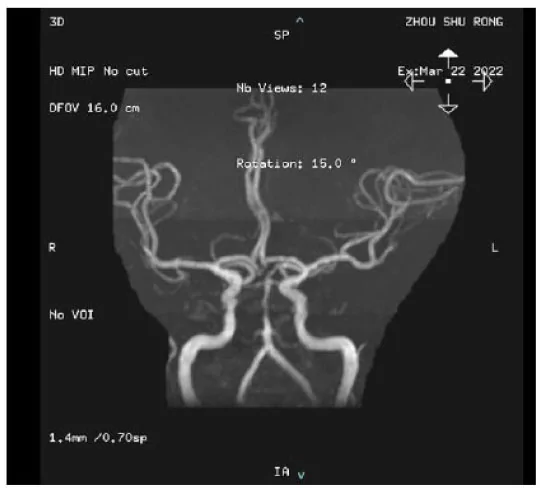
Brain MRA from March 22, 2022, indicates a slight narrowing of the lumen in the basilar artery and arteriosclerosis of the cerebral arteries.

**Fig. (4):**
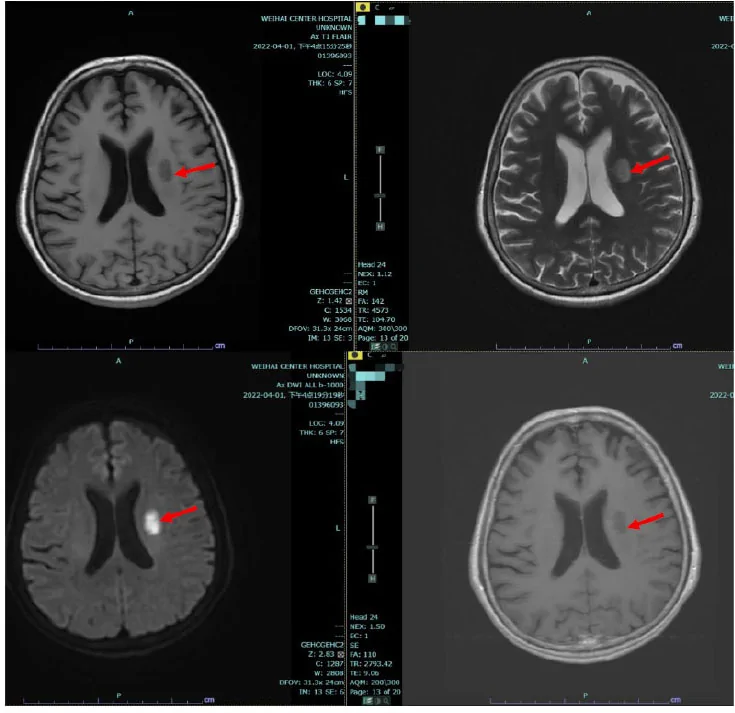
Brain MRI from April 1, 2022, shows patchy areas of prolonged T1 and T2 signal intensities in the left basal ganglia, with high signal on DWI and no significant enhancement on T1 post-contrast.

Laboratory investigations on admission showed normal results for complete blood count, erythrocyte sedimentation rate, urine analysis, stool analysis, liver function tests, biochemistry, and lipid profile. Rheumatoid factor, ACE, and IgG4 antibodies were within normal limits, with a normal anti-double-stranded DNA (anti-dsDNA) level. However, the ANA titer was 1:320, and the ANA spectrum indicated highly elevated anti-SS-A antibodies (+++), anti-SS-B antibodies (+), and anti-Ro-52 antibodies (+++). The patient reported a history of dry mouth lasting over a year, which she attributed to outdoor manual labor, although she had not been concerned about it. CSF analysis revealed a glucose level of 5.32 mmol/L, total protein of 282 mg/L, chloride of 118 mmol/L, and a white blood cell count of 14×10^6^/L. The video EEG showed no abnormalities, and the ECG indicated a sinus rhythm with left-axis deviation. Chest CT revealed increased pulmonary markings and aortic calcification. Brain MRI on March 22, 2022, suggested a fresh infarction in the left basal ganglia region, along with bilateral lacunar infarctions (Fig. **2**). Magnetic Resonance Angiography (MRA) indicated mild local narrowing of the basal arteries and cerebral artery sclerosis (Fig. **3**). The ocular staining score (OSS) was 5. Follow-up brain MRI on April 1, 2022, with contrast enhancement, demonstrated a slightly larger abnormal signal in the left basal ganglia region, consistent with the progression of the infarction. Bilateral lacunar infarctions in the basal ganglia region were observed (Fig. **4**). Salivary gland ECT showed impaired function in the left parotid and submandibular glands, while the intake and excretion function of the right parotid and submandibular glands remained normal.

**Fig. (5):**
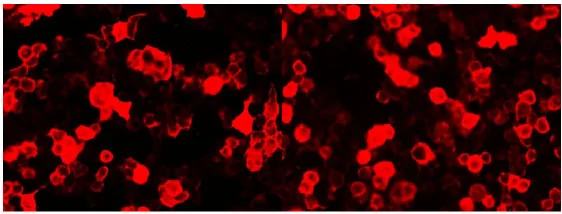
Autoimmune encephalitis antibody profile detection by indirect immunofluorescence method using cell-based assay (CBA) × 200. Through the red light channel of ffuorescence microscopy, a distinct red ffuorescence can be observed in the cell membrane of positive samples. (**A**) LGI1 antibodies positive in the serum. (**B**) LGI1 antibodies positive in the CSF.

Serum autoimmune encephalitis panel showed LGI1 antibody titers of 1:100, confirmed in CSF (conducted at Jinan Jinyu Medical Laboratory) (Fig. **5**). Based on these findings, a diagnosis of anti-LGI1 encephalitis, Sjögren's syndrome, and acute cerebral infarction was considered. Treatment commenced with intravenous immunoglobulin (25 g daily for 5 days), followed by methylprednisolone (1000 mg for 3 days, 500 mg for 3 days, and 250 mg for 3 days). Additionally, aspirin (0.1 g daily), clopidogrel (75 mg daily), and atorvastatin calcium tablets (20 mg daily) were administered. After 9 days of steroid therapy, the patient's right-sided weakness and cognitive impairment improved significantly, with a MoCA score of 21 and negative findings on neurological examination. Muscle strength in the right limbs was restored to 5/5. Oral prednisolone was tapered to 80 mg, and methylprednisolone was discontinued. The patient was prescribed mycophenolate mofetil (0.75 g twice daily), and antiplatelet and anti-atherosclerosis medications were discontinued before discharge.

**Fig. (6):**
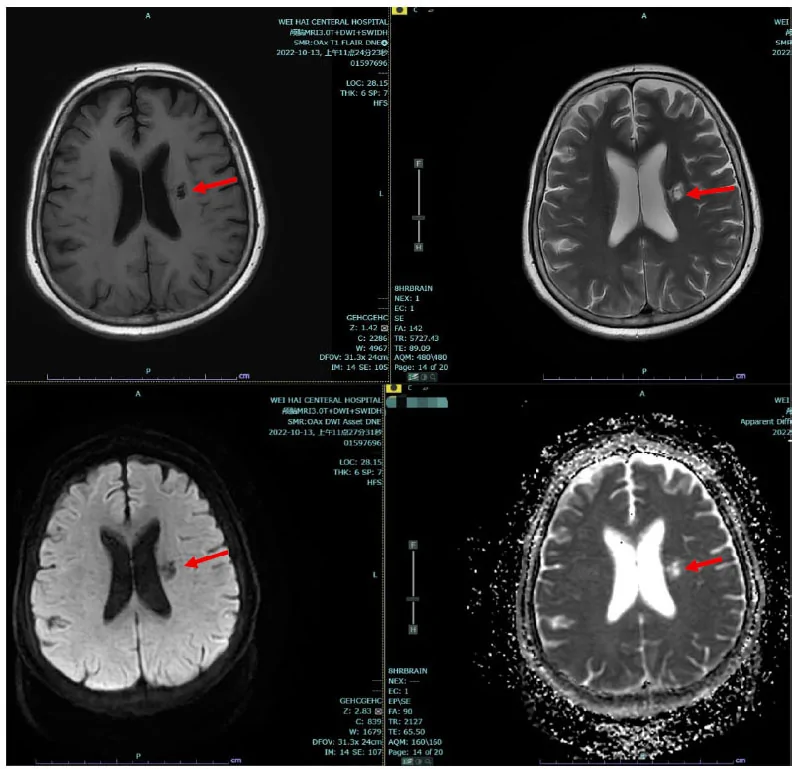
Brain MRI from October 13, 2022, shows patchy areas of prolonged T1 and T2 signal intensities in the left basal ganglia, with low signal on DWI and high signal on FLAIR.

**Fig. (7):**
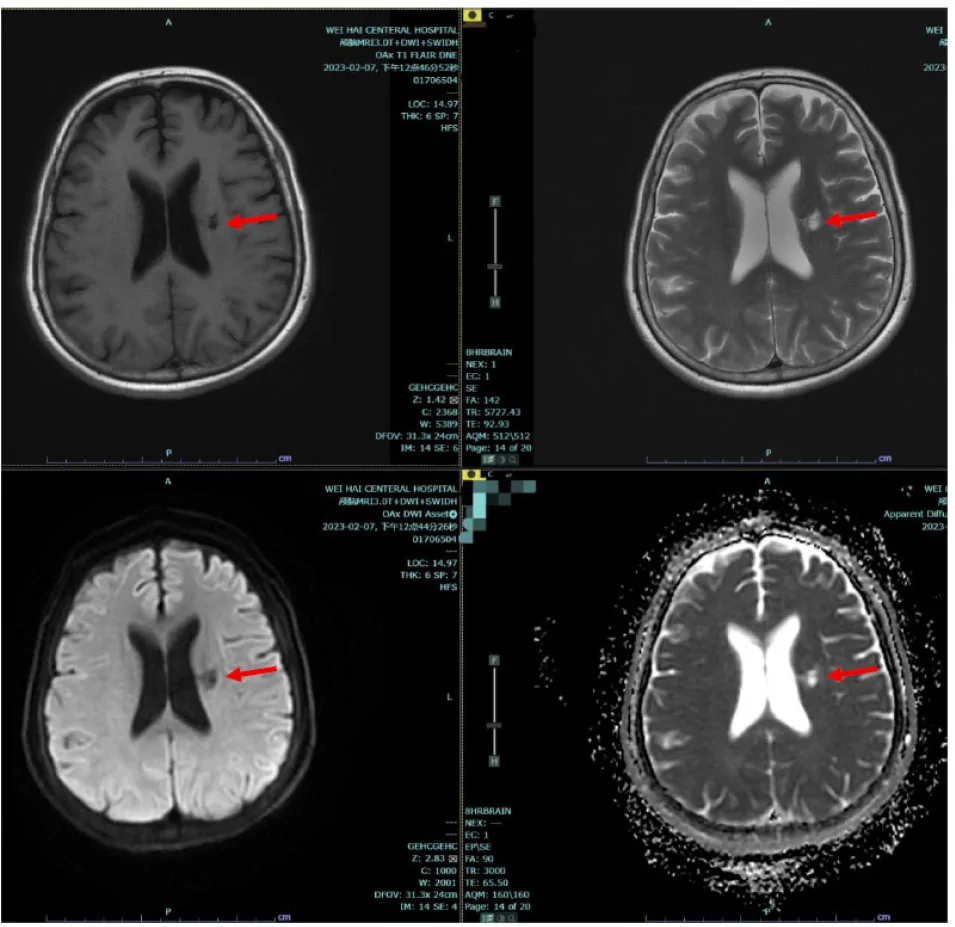
Brain MRI from February 7, 2023, shows patchy areas of prolonged T1 and T2 signal intensities in the left basal ganglia, with low signal on DWI and high signal on FLAIR.

Following discharge, the patient was prescribed oral methylprednisolone at 60 mg daily, with a tapering schedule of one tablet per week until discontinuation. Mycophenolate mofetil was continued at 0.75 g twice daily. Six months later, the patient underwent a follow-up examination, showing complete resolution of clinical symptoms and no neurological signs. Repeat brain MRI revealed an old cerebral infarction in the left basal ganglia region (Fig. **6**), with reduced LGI1 antibody titers (1:10). ANA spectrum indicated elevated levels of anti-SS-A antibodies (++), anti-SS-B antibodies (+), and anti-Ro-52 antibodies (++). The patient continued oral mycophenolate mofetil at a dose of 0.75 g twice daily. At the one-year follow-up visit, the patient remained asymptomatic, with repeat MRI showing the same old cerebral infarction in the left basal ganglia region (Fig. **7**). Maintenance therapy with mycophenolate mofetil (0.75 g BID) was continued. Currently, the patient continues to be monitored under ongoing follow-up.

## 3 DISCUSSION

Anti-leucine-rich glioma inactivated-1 (LGI1) antibody-associated encephalitis is an autoimmune encephalitis mediated by anti-LGI1 antibodies, and is one of the primary subtypes of autoimmune encephalitis, more commonly found in middle-aged and elderly men [1]. LGI1 is a secreted neuronal protein located in the synaptic cleft, primarily expressed in the temporal cortex and hippocampus. It forms a trans-synaptic complex with presynaptic ADAM23 and Kv1.1 potassium channels, as well as postsynaptic ADAM22 and AMPA receptors (AMPAR). LGI1 antibodies affect the interaction between ADAM23 and ADAM22, leading to dysfunction of the presynaptic voltage-gated K^+^ channels and postsynaptic AMPARs, thereby impairing synaptic transmission [4]. Clinical manifestations of LGI1 antibody-associated encephalitis [5] mainly include seizures, impaired memory function (primarily recent memory loss), and psychiatric disorders. Additionally, some patients present with autonomic nervous system dysfunction, sleep disorders, speech disorders, and refractory hyponatremia. Acute or early findings on cranial MRI typically show increased signals in the hippocampus, amygdala, temporal lobes, insular lobes, and cingulate gyrus on T2-weighted and diffusion-weighted imaging sequences, often with unilateral or symmetric swelling [6]. A slight increase in CSF protein and lymphocyte count may be observed in some patients; however, the detection of LGI antibodies in serum and CSF remains the most specific marker for diagnosis [7]. In this case, the middle-aged female patient had a relapsing course characterized by delayed response, altered mental status, and right-sided weakness. MRI findings showed abnormal signals in the bilateral hippocampal regions, and both serum and CSF tested positive for LGI1 antibodies, confirming the diagnosis of LGI1 antibody-associated encephalitis.

Primary Sjögren's syndrome [8] is a chronic inflammatory autoimmune disease that primarily affects the exocrine glands. It typically presents with an insidious onset, characterized by hallmark symptoms of dry mouth and dry eyes. Extraglandular manifestations can involve multiple organ systems, including neurological, vascular, and musculoskeletal systems. Neurological involvement in Sjögren's syndrome is varied and may include peripheral neuropathy (commonly symmetric limb numbness and weakness), autonomic dysfunction (such as orthostatic hypotension, tachycardia, and gastrointestinal disturbances), and central nervous system complications, which can range from cognitive impairment to ischemic stroke, headaches, and demyelinating diseases [2]. In this case, the patient reported a history of dry eyes and dry mouth, with markedly elevated levels of anti-SS-A and anti-Ro-52 antibodies, and a conjunctival corneal staining score of 5. Combined with salivary gland CT results, the diagnosis of primary Sjögren's syndrome was confirmed.

LGI1 antibody-associated encephalitis combined with primary Sjögren's syndrome is a clinically rare occurrence. Zhao *et al* [3] have reported that patients with autoimmune encephalitis (AE) may concurrently have systemic autoimmune diseases, including Hashimoto's thyroiditis, systemic lupus erythematosus, allergic purpura, Sjögren's syndrome, and chronic urticaria. Among them, patients with LGI1 antibody-associated encephalitis are more likely to have concurrent systemic autoimmune diseases compared to other autoimmune encephalitis. This is attributed to the strong correlation between LGI1 antibody-associated encephalitis and the expression of human leukocyte antigen (HLA) class II genes [9-11]. HLA genes are critical in the pathogenesis of autoimmune diseases of the nervous system as they encode cell surface antigen-presenting proteins directly involved in immune responses. Genetic studies [12, 13] have found associations between HLA class II alleles and many systemic autoimmune diseases. Cruz's study [14] identified HLA-DQA1*0501, HLA-DQB1*0201, and HLADRB1*0301 as genetic risk factors for primary Sjögren's syndrome, while Reveille's study [15] found that alleles of HLA-DQB1 and HLA-DQA1 are closely associated with anti-Ro antibody-positive Sjögren's syndrome patients. A study from China revealed a close genetic association between LGI1 antibody-associated encephalitis and alleles, such as DRB1*03:01 or DQB1*02:01, as well as the extended DRB1*03:01-DQB1*02:01 haplotype [16]. These studies suggest that LGI1 antibody-associated encephalitis and Sjögren's syndrome may share common susceptibility genes, such as DRB1*03:01 and DQB1*0201, indicating a possible common immunogenetic background. Under specific autoimmune conditions, these two diseases may co-occur, highlighting the importance of genetic predisposition in their simultaneous manifestation.

In addition, the patient presented with acute-onset right-sided weakness. A cranial MRI showed high signal intensity on diffusion-weighted imaging (DWI) and low signal intensity on the diffusion coefficient (ADC), with no significant enhancement on contrast-enhanced scans, confirming the diagnosis of acute cerebral infarction. However, the combination of LGI1 encephalitis, Sjögren's syndrome, and acute cerebral infarction is clinically rare. The patient had no history of hypertension, diabetes, hyperlipidemia, or other common cerebrovascular disease risk factors, and the infarction was not related to large artery atherosclerosis, suggesting the possible association with autoimmune encephalitis and Sjögren's syndrome. Few cases of autoimmune encephalitis combined with cerebral infarction have been reported. McGinley *et al*. [17] reported a case of LGI1 antibody-associated encephalitis combined with subacute cerebral infarction, while Hu *et al*. [18] reported a case of anti-glutamic acid decarboxylase antibody-associated encephalitis combined with cerebral infarction.

The proposed mechanism may involve immune-mediated endothelial inflammation induced by LGI1 antibodies, leading to vascular occlusion. Concurrently, autoimmune encephalitis may upregulate antibodies that mediate vascular injury, associated with systemic autoimmune diseases, and in this case, the patient coincidentally had Sjögren's syndrome, which is a significant cause of secondary central nervous system vasculitis, with the pathological mechanism of vascular damage likely involving mononuclear cell-mediated cerebral vasculitis, primarily affecting small vessels in the subcortical white matter and periventricular regions, manifested by inflammatory cell infiltration, vascular wall fibrosis, and occlusion leading to acute cerebral infarction [19]. Recent studies have further demonstrated that Sjögren’s syndrome may also involve large vessels, including the internal carotid artery and the middle cerebral artery, and can result in recurrent watershed infarctions. High-resolution magnetic resonance vessel wall imaging (HRMR-VWI) has emerged as a valuable tool for visualizing inflammatory changes in the walls of large cerebral arteries, providing improved diagnostic insight into CNS vasculitis associated with Sjögren’s syndrome [20].

For the treatment of LGI1 antibody-associated encephalitis, immunotherapy is the first-line approach. Commonly used therapies include glucocorticoids, intravenous immunoglobulin, and plasma exchange. For patients who do not respond to these treatments, second-line therapies, such as cyclophosphamide and rituximab, may be considered [21]. For patients with comorbid epilepsy, a ketogenic diet is recommended to improve seizure control. The proposed mechanisms may involve ketone bodies and polyunsaturated fatty acids acting as mediators to modulate neuronal excitability and confer neuroprotection. Additionally, ketone bodies have been shown to suppress the assembly of inflammasomes, thereby having anti-inflammatory effects [22].In this case, the patient was promptly treated with intravenous methylprednisolone pulse therapy following the diagnosis of LGI1 antibody-associated encephalitis, resulting in significant symptom improvement. Oral administration of mycophenolate mofetil was later used for ongoing immunotherapy. The patient was followed for one year and has now experienced complete recovery, with no residual sequelae. Thus, the patient’s favorable clinical outcome may be related to the prompt administration of immunotherapy in the early disease stage and the presence of a low level of pathogenic antibodies [23].

## CONCLUSION

In conclusion, the co-occurrence of LGI1 antibody-associated encephalitis and primary Sjögren's syndrome is rare, and the exact mechanisms of this comorbidity remain unclear. The simultaneous occurrence of acute cerebral infarction is uncommon, and its pathogenesis likely involves immune-related vasculitis. This case contributes to a better understanding of the relationship between LGI1 antibody-associated encephalitis, Sjögren's syndrome, and cerebral infarction. Further clinical studies are necessary to explore potential pathogenic mechanisms in greater detail. Nonetheless, this study still has limitations: First, the sample homogeneity of single-center case reports and retrospective design limit the generalizability of conclusion, with risks of selection bias and chance associations; second, the lack of longitudinal monitoring of autoantibody production makes it difficult to establish a causal relationship between LGI1 antibodies and Sjögren's syndrome; Third, the mechanism of vasculitis-induced cerebral infarction lacks histological evidence, and reliance on indirect laboratory indicators introduces diagnostic bias. Future multicenter studies should incorporate cerebrospinal fluid cytokine profiling, vascular endothelial function testing, and neuropathological validation of the immune-mediated vascular injury hypothesis.

## AUTHORS’ CONTRIBUTIONS

The authors confirm their contributions to the paper as follows: Study conception and design: DJ; data collection: XZ; analysis and interpretation of results: YL; Writing - Original Draft Preparation: PX; Writing - Reviewing and Editing: YB; Writing the Paper: JW. All authors reviewed the results and approved the final version of the manuscript.

## Data Availability

The data support the findings of this study are available from the corresponding author upon reasonable request.

## LIST OF ABBREVIATIONS

FBDS: Faciobrachial Dystonic Seizures
CSF: Cerebrospinal Fluid
MoCA: Montreal Cognitive Assessment
MRA: Magnetic Resonance Angiography
OSS: Ocular Staining Score
Anti-LGI1: Anti-Leucine-rich Glioma Inactivated-1
AE: Autoimmune Encephalitis
HLA: Human Leukocyte Antigen
DWI: Diffusion-Weighted Imaging

## STANDARDS OF REPORTING

CARE guidelines were followed.
